# Low inter-observer agreement among experienced shoulder surgeons assessing overstuffing of glenohumeral resurfacing hemiarthroplasty based on plain radiographs

**DOI:** 10.1186/s13018-018-1008-6

**Published:** 2018-11-27

**Authors:** Nicolai Sandau, Stig Brorson, Bo S. Olsen, Anne Kathrine Sørensen, Steen L. Jensen, Kim Schantz, Janne Ovesen, Jeppe V. Rasmussen

**Affiliations:** 1 Department of Orthopedic Surgery, Herlev & Gentofte Hospital, Herlev Ringvej 75, 2730 Herlev, Denmark; 2 Department of Orthopedic Surgery, Aalborg University Hospital, Højgårdsvej 11, 9640 Farsø, Denmark; 3 Department of Orthopedic Surgery, Zealand University Hospital, Lykkebækvej 1, 4600 Køge, Denmark; 4 Department of Orthopedic Surgery, Aarhus University Hospital, Palle Juul Jensens Boulevard 99, 8000 Aarhus, Denmark

## Abstract

**Background:**

In a clinical setting, a visual evaluation of post-implant radiographs is often used to assess the restoration of glenohumeral joint anatomy after resurfacing hemiarthroplasty and is a part of the decision-making process, in combination with other parameters, when evaluating patients with inferior clinical results. However, the reliability of this method of visual evaluation has not been reported. The aim of this study was to investigate the inter- and intra-observer agreement among experienced shoulder surgeons assessing overstuffing, implant positioning, and size following resurfacing hemiarthroplasty using plain standardized radiographs.

**Methods:**

Six experienced shoulder surgeons independently classified implant inclination, size of the implant and if the joint seemed overstuffed, in 219 cases of post-implant radiographs. All cases were classified twice 3 weeks apart. Only radiographs with an anterior-posterior projection with a freely visible joint space were used. Non-weighted Cohen’s kappa values were calculated for each coder pair and the mean used as an estimate of the overall inter-observer agreement.

**Results:**

The overall inter-observer agreement for implant size (kappa, 0.48 and 0.41) and inclination angle was moderate in both rounds (kappa, 0.46 and 0.44), but only a fair agreement was found concerning the evaluation for stuffing of the joint (kappa, 0.24 and 0.28). Intra-observer agreement for implant size and stuffing ranged from fair to substantial while the agreement for inclination was moderate to substantial.

**Conclusions:**

Our results indicate that a visual evaluation of plain radiographs may be inadequate to evaluate overstuffing, implant positioning, and size following resurfacing hemiarthroplasty using plain standardized radiographs. Future studies may contribute to elucidate whether reliability increases if consensus on clear definitions and standardized methods of evaluation is made.

## Introduction

Resurfacing hemiarthroplasty (RHA) was developed to restore normal anatomy, and with a bone-preserving design and short operation time, it has often been preferred for the treatment of glenohumeral osteoarthritis [4, 10, 26, 27]. Some studies have reported good functional outcome and a low rate of revision [14–16, 22, 23, 28, 31], while others report a poor functional outcome and a high risk of revision [7, 8, 10, 11, 17, 24]. This has led to concerns that RHA may not adequately restore humeral anatomy [19].

Studies evaluating the restoration of glenohumeral joint anatomy following RHA have been conflicting. Some report that RHA restores humeral head anatomy [9, 18, 30] while others report increased lateral glenohumeral offset (LGHO) [14, 17–20, 28], displacement of the center of rotation [3], increased humeral head size [27], and a tendency to place the implant in varus [14, 18]. Despite a lack of a clear definition, the term overstuffing has also been widely used in the literature as a possible cause of persistent pain or a poor functional outcome following RHA [1–3, 6, 18, 19, 21, 25–27, 29, 30, 32]. Plain radiographs are the most common image modality when evaluating patients with a poor functional outcome or persistent pain following shoulder arthroplasty [5]. In a clinical setting, a visual evaluation of post-implant radiographs is often used to assess the restoration of glenohumeral joint anatomy after RHA and is, in combination with other parameters, a part of the decision-making process, when evaluating patients with inferior clinical results. However, the reliability of this method of visual evaluation has not been elucidated.

Therefore, the aim of this study was to investigate the inter- and intra-observer agreement among experienced shoulder surgeons assessing overstuffing, implant inclination, and size following RHA using plain standardized radiographs.

## Materials and methods

Three hundred eighty-two patients treated with primary RHA at one of four Danish university hospitals between January 2006 and December 2013 were retrospectively identified using the Danish Shoulder Arthroplasty Registry. Post-implant radiographs were digitally collected for each patient and evaluated for eligibility by the last author who was not an observer. Only cases with radiographs in an anterior-posterior projection with a freely visible joint space were included. One hundred sixty-three cases were excluded due to poor quality leaving 219 cases to be included in the study.

Six experienced shoulder surgeons, with a mean work experience of more than 10 years and a surgical volume of more than 50 shoulder arthroplasty procedures per year, were chosen as observers. The observers were all employed at one of the four hospitals providing radiographs. All radiographs were anonymized by digitally cropping out any patient data and hospital affiliation printed on the radiographs. The file names of the digital radiographs were then randomized using Excel (Microsoft, Redmond, Washington) before being sent to the observers for classification.

To ensure that all observers used similar visual characteristics of the categories 22 radiographs (10%) were retrieved by the same surgeon who evaluated the radiographs for eligibility and digitally presented to the group of observers. The group of observers then collectively chose one radiograph per classification they regarded as exemplifying the following; too small implant size, too large implant size, overstuffed, understuffed, valgus positioning, and varus positioning. This was done 3 weeks before the first classification round. The final results were calculated and analyzed both with and without the 22 cases used for the consensus classifications.

The observers independently classified all radiographs on two occasions 3 weeks apart. The order of the radiographs was randomized between the classification rounds. In both classification rounds, the observers were asked to evaluate the radiographs in terms of (1) inclination angle (varus, valgus, or anatomical), (2) size of RHA in the relation to the patient’s anatomy (too large, too small, or anatomical), and (3) stuffing of the joint (overstuffed, understuffed, or anatomical). The observers registered their evaluations for each radiograph in a separate spreadsheet for each classification round. The observers were not given any additional information about the patients, and they were not allowed to use any measurement tools in their evaluation. It was stressed that all radiographs should be evaluated also if the observer found the radiographs of insufficient quality.

### Statistics

The percentage of total observed agreement was calculated as the proportion of cases where all observers agreed upon the same classification. Non-weighted Cohen’s kappa values were calculated for each coder pair and the mean used as an estimate of the overall inter-observer agreement. The intra-observer agreement was calculated as the non-weighted Cohen’s kappa values for each individual observer between the two classification rounds. The agreement was calculated for inclination, implant size, and stuffing of the joint. Inter- and intra-observer agreement was calculated for both the 197 cases excluding the consensus cases and for the pooled answers of all 219 cases. Including the consensus, cases did not change the results or alter the conclusions of the present study. Therefore, the results are presented for all 219 cases. Kappa values were qualitatively interpreted using the ranges proposed by Landis and Koch with values less than 0 indicating poor agreement, 0.00–0.20 slight agreement, 0.21–0.40 fair agreement, 0.41–0.60 moderate agreement, 0.61–0.80 substantial agreement, and 0.81–1.00 excellent agreement [13].

Percentage of agreement and kappa values were calculated using the “irr” package and bootstrapping of the confidence intervals using the “boot” package in R statistical software version 3.3.2 (R foundation for statistical Computing, Vienna, Austria).

### Compliance with ethical standards

The present study has been approved by the Danish Health and Medicines Authority (case no. 3-3013-1103/1/, 28/7-2015) and the Danish data protection agency (03663. ID no. HEH-2015-037, 20/04-2015).

## Results

The percentage of inter-observer agreement ranged from 17.8 to 43.4% (Table 1).

**Table 1:** Percentage of inter-observer agreement

	Implant size	Inclination	Stuffing
Round 1 (%)	40.6	43.4	17.8
Round 2 (%)	38.4	43.4	21.5

The overall inter-observer agreement for implant size and inclination was moderate, and for the stuffing category, the agreement was fair (Table 2).

**Table 2:** Mean kappa values for overall inter-observer agreement

	Implant size	Inclination	Stuffing
Round 1 (95% CI)	0.48 (0.43–0.55)	0.46 (0.39–0.53)	0.24 (0.20–0.29)
Round 2 (95% CI)	0.41 (0.35–0.47)	0.44 (0.37–0.52)	0.28 (0.24–0.34)

*CI* confidence interval

The percentage of intra-observer agreement ranged from 68.0 to 90.4% (Table 3).

**Table 3:** Percentage of intra-observer agreement

	Observer no.					
	1	2	3	4	5	6
Implant size (%)	87.7	82.6	79.5	69.4	87.7	86.3
Inclination (%)	87.7	81.7	79.0	89.0	85.5	90.4
Stuffing (%)	68.0	80.8	84.9	79.9	78.1	83.6

Intra-observer agreement for implant size and stuffing ranged from fair to substantial while the agreement for inclination was moderate to substantial (Table 4).

**Table 4:** Kappa values for intra-observer agreement

	Observer no.					
	1	2	3	4	5	6
Implant size	0.75	0.70	0.60	0.41	0.72	0.57
Inclination	0.66	0.70	0.56	0.61	0.69	0.75
Stuffing	0.31	0.56	0.58	0.58	0.56	0.64

**Fig. 1:**
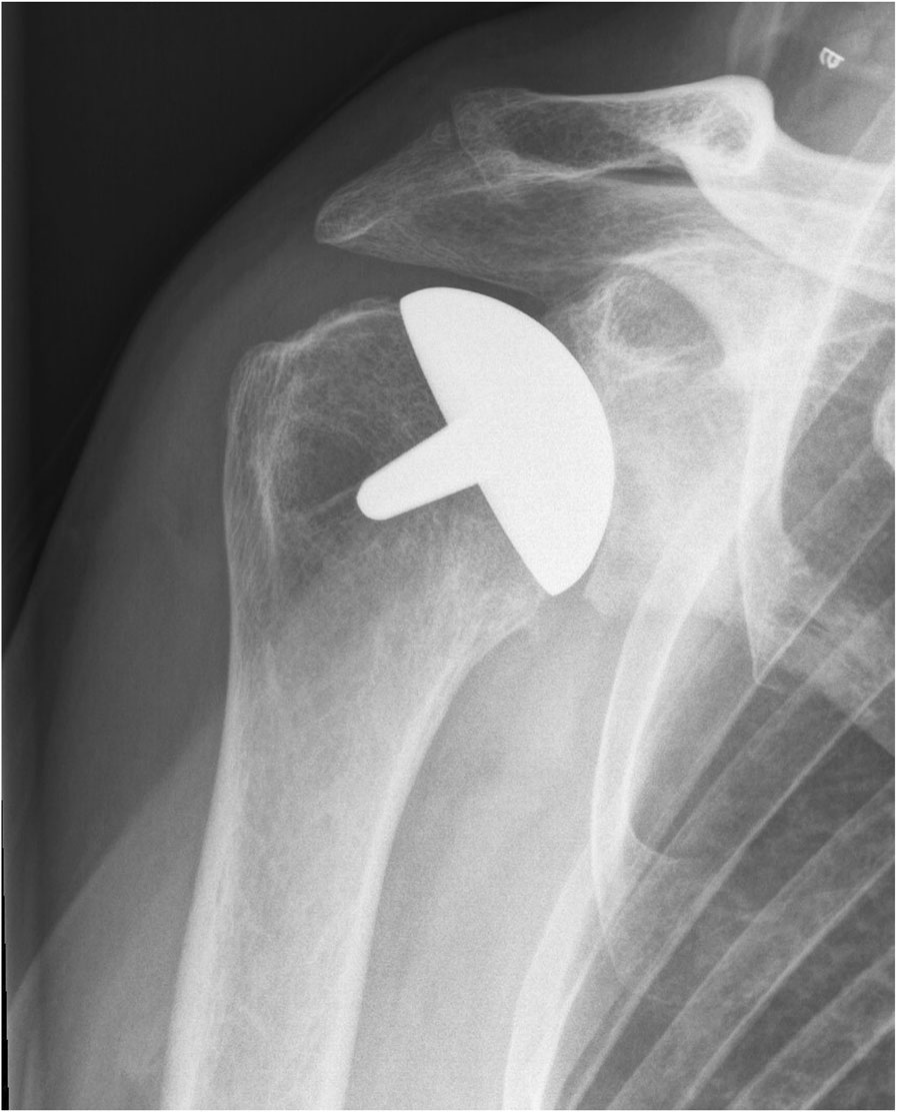
Post-implant AP radiograph where all observers classified the size, inclination, and stuffing of the joint as anatomical

**Fig. 2:**
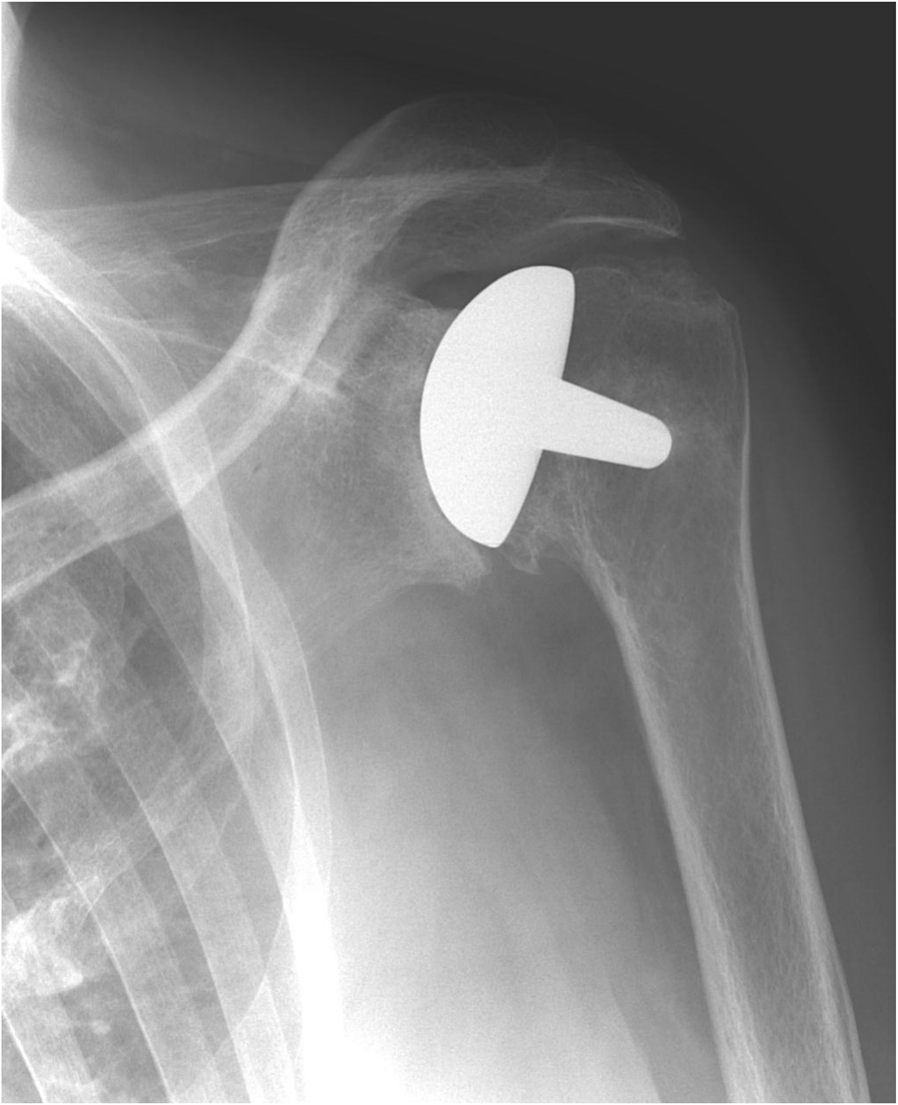
Post-implant AP radiograph where all observers classified the joint as overstuffed

**Fig. 3:**
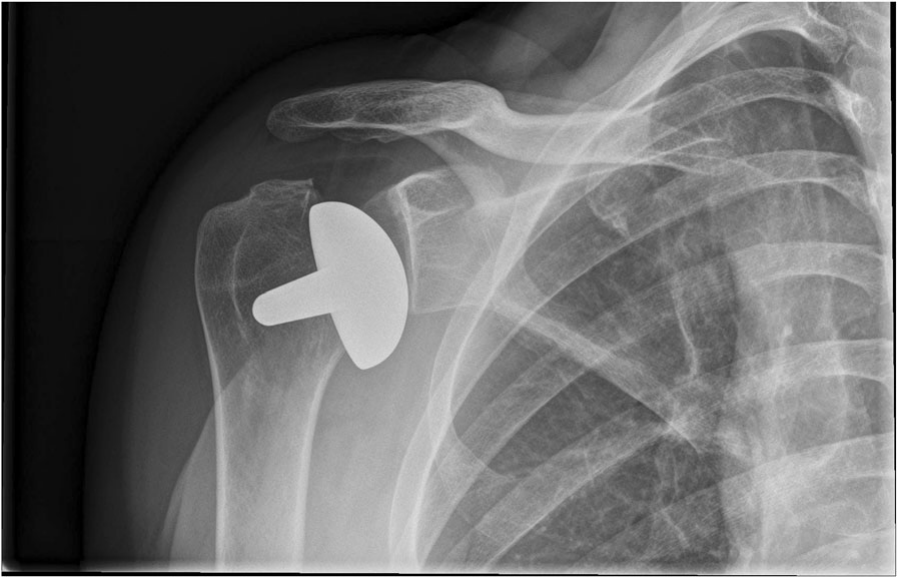
Post-implant AP radiograph where all observers classified the inclination as being in varus

**Fig. 4:**
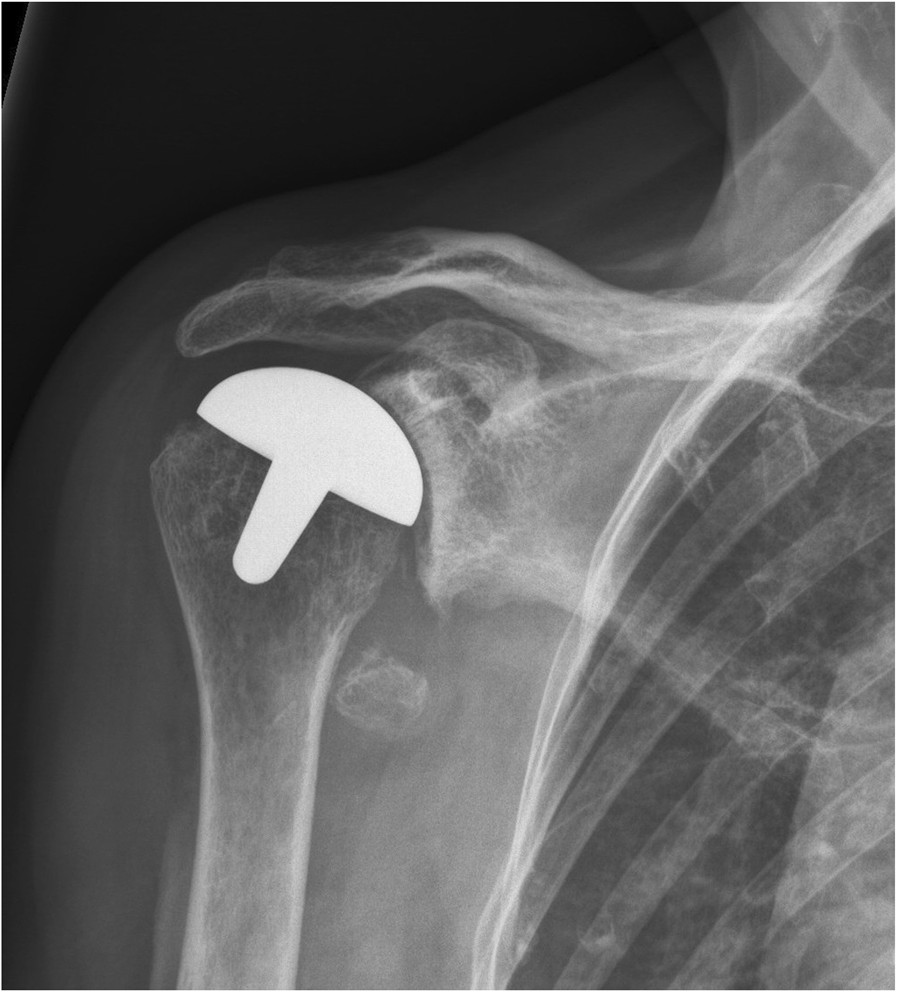
Post-implant AP radiograph where all observers classified the inclination as being in valgus

**Fig. 5:**
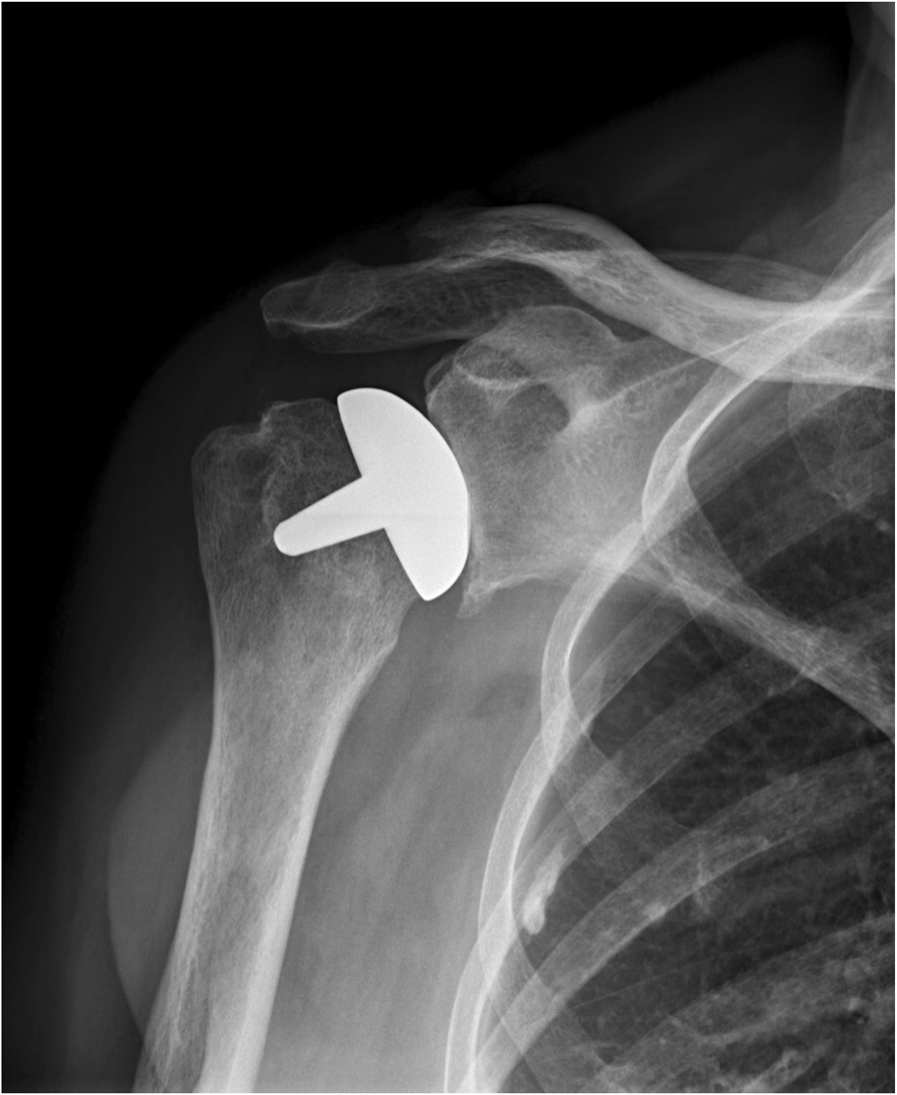
Post-implant AP radiograph where all observers classified the size of the implant as too small

**Fig. 6:**
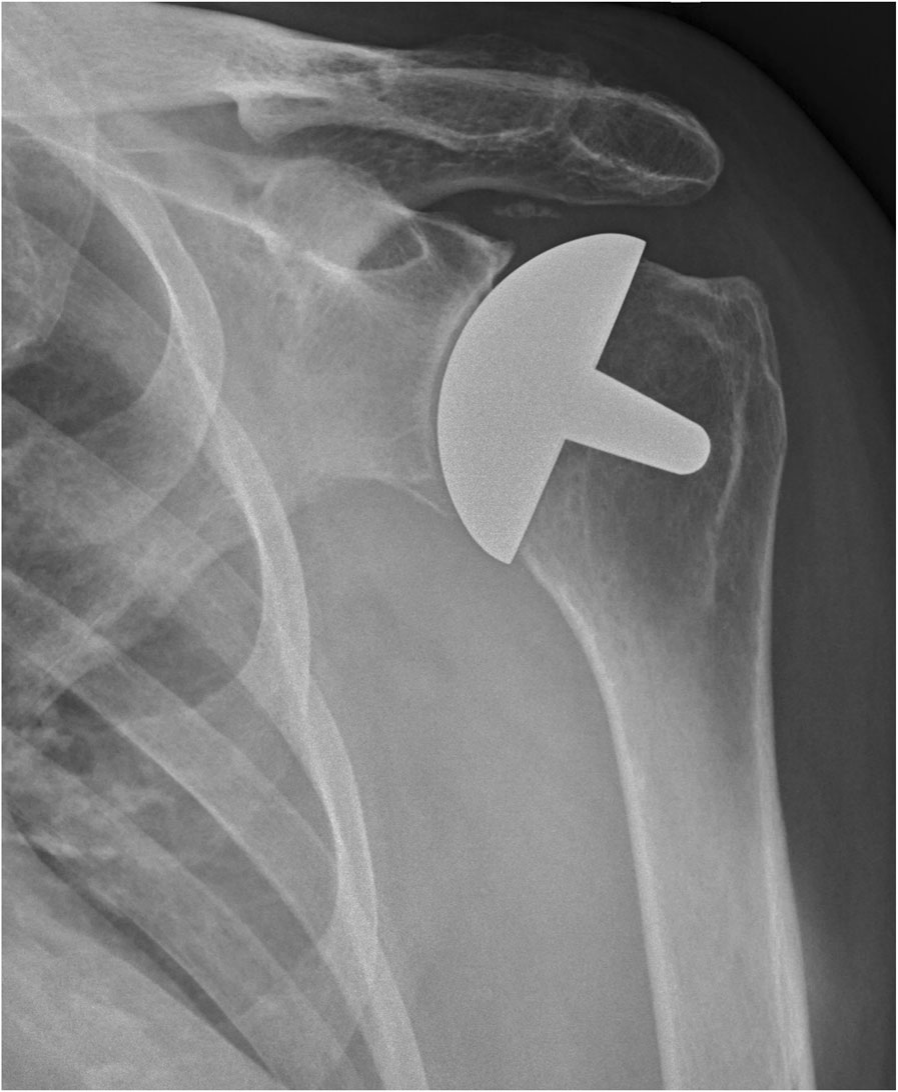
Post-implant AP radiograph where all observers classified the size of the implant as too large

Examples of radiographs where the observers all agreed upon a classification is given in Figs. 1, 2, 3, 4, 5, and 6.

## Discussion

We observed moderate overall inter-observer agreement for the evaluation of implant size and inclination, but only fair agreement for overstuffing of the joint among experienced shoulder surgeons. Thus, indicating that a visual evaluation of plain radiographs may be inadequate to evaluate overstuffing, implant positioning, and size following RHA using plain standardized radiographs.

We observed moderate overall inter-observer agreement for the evaluation of size and inclination but only fair agreement for overstuffing of the joint among experienced shoulder surgeons.

With this study, we wanted to investigate if there was a uniform visual recognition pattern among shoulder surgeons for evaluating overstuffing, implant inclination, and size of the implant following RHA using plain standardized radiographs. Only a moderate inter-observer agreement was observed for the classification of implant size and inclination. Humeral head size and implant inclination based on plain radiographs following RHA are often reported in the literature [1, 14, 18, 27, 30]. However, data is lacking on the reliability of such evaluations following RHA. Our study indicates that a visual evaluation of both implant inclination and implant size might not be a reliable method of assessment.

Furthermore, we only demonstrated a fair agreement between the observers when evaluating overstuffing. We hypothesize that the lower agreement between the observers for the stuffing category is primarily caused by a general lack of clear definition between the observers causing them to rely on their own subjective pattern recognition. The higher intra-observer agreement indicates that the observers individually use their own subjective method to evaluate stuffing between the two classification rounds. However, it seems that their subjective pattern recognitions are different between the observers shown by the low inter-observer agreement. This tendency is also observed when examining the intra- and inter-observer agreements for inclination and implant size although the effect is less marked. This could indicate that a visual evaluation as a method is not sufficiently standardized and is heavily influenced by the surgeons’ own subjective way of pattern recognition, especially apparent regarding overstuffing. Therefore, a more standardized method of defining and measuring overstuffing might aid to increase the intra- and inter-observer agreement.

Multiple definitions of overstuffing following RHA have been used in the literature including a medial deviation of the center of rotation [3], increased LGHO [19, 20], and improper implant size [1, 2, 5, 6, 21, 28], but little data exist on the reliability of these measurements. A recent study by Kadum et al. investigated intra- and inter-observer agreement between four observers measuring LGHO on both computed tomography (CT) images and radiographs. The authors reported excellent inter- and intra-observer agreement when measuring LGHO on CT images. When measuring LGHO on plain radiographs only moderate inter-observer agreement was reported. When comparing measurements from CT images and radiographs, the authors found a tendency to underestimate the LGHO on radiographs with a mean difference of 5 mm [12]. Another study by Thomas et al. also found low inter-observer agreement when measuring LGHO on plain radiographs. This was related to a systematic error with one observer locating the base of the coracoid more medially than the other. The authors concluded that their measurements of LGHO were unreliable [30]. In an attempt to minimize such systematic errors, Stilling et al. created a modified method of measuring LGHO. The measurements were done by a radiologist, and intra-observer agreement was reported as being high but no data on the inter-observer agreement was reported [29]. Based on these previous results, LGHO measured on plain radiographs does not seem like a viable method to evaluate the anatomical reconstruction or as a framework to define overstuffing.

Alolabi et al. used a spherical model mapped to preserved non-articular bone landmarks to assess the anatomical reconstruction of the center of rotation following RHA on pre- and post-implant radiographs. This was based on assessments done by four observers with cases evenly distributed between them, and therefore, no information on the inter-observer agreement was reported [3].

Based on the above, it currently seems that there are no reported methods of reliably assessing overstuffing following RHA, and thereby, no method of defining the term. Our study indicates that a uniform visual recognition pattern regarding overstuffing, implant inclination, and size of the implant following RHA does not exist and that each observer has their own subjective method of pattern recognition, especially regarding overstuffing. Therefore, a visual evaluation does not seem like a reliable method to define and asses overstuffing following RHA.

There are limitations to this study. Firstly, we had to exclude a significant amount of radiographs due to poor quality thereby introducing the possibility of selection bias. However, we hypothesize that had all the collected radiographs been included, we would probably have seen an even lower agreement. In a clinical setting, one would have the possibility of ordering supplemental radiographs if the quality was insufficient for clinical decision-making. Therefore, we believe the exclusion of radiographs without a visible joint space makes our results applicable to a clinical setting. Secondly, there is a possibility of recall bias when using the same radiographs between the two classification rounds. We tried to minimize this by including a high number of radiographs, randomizing the radiographs between classification rounds, and placing the two classification rounds 3 weeks apart. Despite this, it is possible that some of the observers could remember their answers for specific radiographs, thereby primarily affecting the intra-observer agreement. Thirdly, the external validity of the study may be questioned in terms of a lack of generalizability to less experienced observers as all the observers in the current study were experienced with more than 10 years of experience in shoulder surgery. Despite this, we decided to only include experienced shoulder surgeons as they are most likely to be involved in the decision-making process regarding patients with a poor functional outcome or persistent pain.

## Conclusions

The present study only found a fair inter-observer agreement between experienced shoulder surgeons assessing stuffing of the shoulder joint and moderate inter-observer agreement when assessing the inclination and implant size based on plain radiographs. Thus, indicating that a visual evaluation of plain radiographs may be inadequate to evaluate overstuffing, implant positioning, and size following RHA using plain standardized radiographs. Future studies may contribute to elucidate whether reliability increases if consensus on clear definitions and standardized methods of evaluation can be made.
